# Computer vision uncovers three fundamental dimensions of levodopa-responsive motor improvement in Parkinson’s disease

**DOI:** 10.1038/s41531-025-00999-w

**Published:** 2025-05-28

**Authors:** Florian Lange, Diego L. Guarin, Esther Ademola, Dalia Mahdy, Gabriela Acevedo, Thorsten Odorfer, Joshua K. Wong, Jens Volkmann, Robert Peach, Martin Reich

**Affiliations:** 1 Department of Neurology, University of Würzburg, Würzburg, Germany; 2 Movement Estimation and Analysis Laboratory, Department of Applied Physiology and Kinesiology, University of Florida, Gainesville, FL USA; 3 Fixel Institute for Neurological Disease, College of Medicine, University of Florida, Gainesville, FL USA; 4 Crayton Pruitt Family Department of Biomedical Engineering, University of Florida, Gainesville, FL USA; 5 Department of Neurology, College of Medicine, University of Florida, Gainesville, FL USA

**Keywords:** Computational neuroscience, Neurological disorders, Parkinson's disease

## Abstract

We developed VisionMD, an AI computer vision platform, analyzing over 1200 clinical videos of Parkinson’s patients’ hand movements across 13 years. This large-scale, markerless analysis identified three kinematic domains (speed, consistency, timing/scale) reliably improved by levodopa. Our method offers objective, quantitative motor assessment, reducing subjectivity and enhancing reproducibility compared to traditional scales.

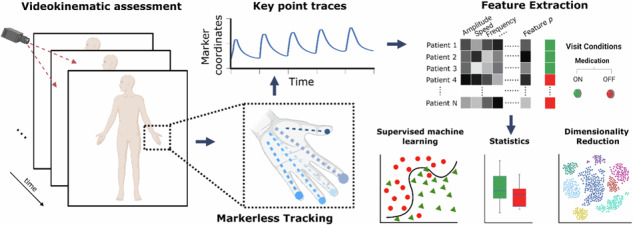

## Introduction

The clinical assessment of motor function is central to the diagnosis and management of movement disorders like Parkinson’s disease (PD). Yet, achieving accurate and objective measures of motor symptoms remains a key challenge in PD management^1^. Traditional clinical scales, such as the Movement Disorder Society Unified Parkinson’s Disease Rating Scale (MDS-UPDRS), rely on clinician judgment, rendering them susceptible to inter- and intra-rater variability and limiting their sensitivity to subtle treatment effects^1^. Even among movement-disorder experts, interrater reliability remains only moderate, and false positives are common, with one in four healthy controls misclassified as having bradykinesia^2^.

Recent advances in artificial intelligence (AI)-based digital biomarkers and video-driven kinematic analyses promise to address these shortcomings by delivering more objective, reproducible, and accessible evaluations of motor function^3–8^. Building on these developments, we have recently introduced VisionMD, an open-source software platform for video-based kinematic analysis, to support efforts towards more standardized and quantifiable clinical assessments^9^. Since fine motor control of the hands is particularly affected by bradykinesia, and tasks like finger tapping and hand movements are standard components of clinical evaluation, analyzing these specific movements provides a valuable window into overall PD motor impairment and treatment response.

In this study, we illustrate how a digital measurement tool—VisionMD, an open-source, video-based kinematic analysis platform can help address key challenges in digital interventions. Unlike many existing approaches, VisionMD requires no wearable markers and can be applied retrospectively to heterogeneous clinical video archives. Over 1200 clinical videos of 154 PD patients performing standardized hand opening and finger tapping tasks were analyzed under both ON and OFF levodopa conditions. This rich, real-world dataset—collected over a 13-year period—allowed us to rigorously characterize the kinematic response to dopaminergic therapy.

Through sparse principal component analysis (PCA) of the kinematic features, we uncovered three stable domains—movement speed, consistency, and movement timing & scale—that reliably improve with levodopa administration. Movement Speed characterizes the speed of alternating movements, Consistency captures their stability and uniformity across repeated cycles, and Movement Timing & Scale describes how movements are patterned and sequenced over time. Together, these domains represent fundamental aspects of bradykinesia-related motor impairment and highlight distinct, quantifiable signatures of levodopa-responsible motor execution deficits.

**Fig. 1:**
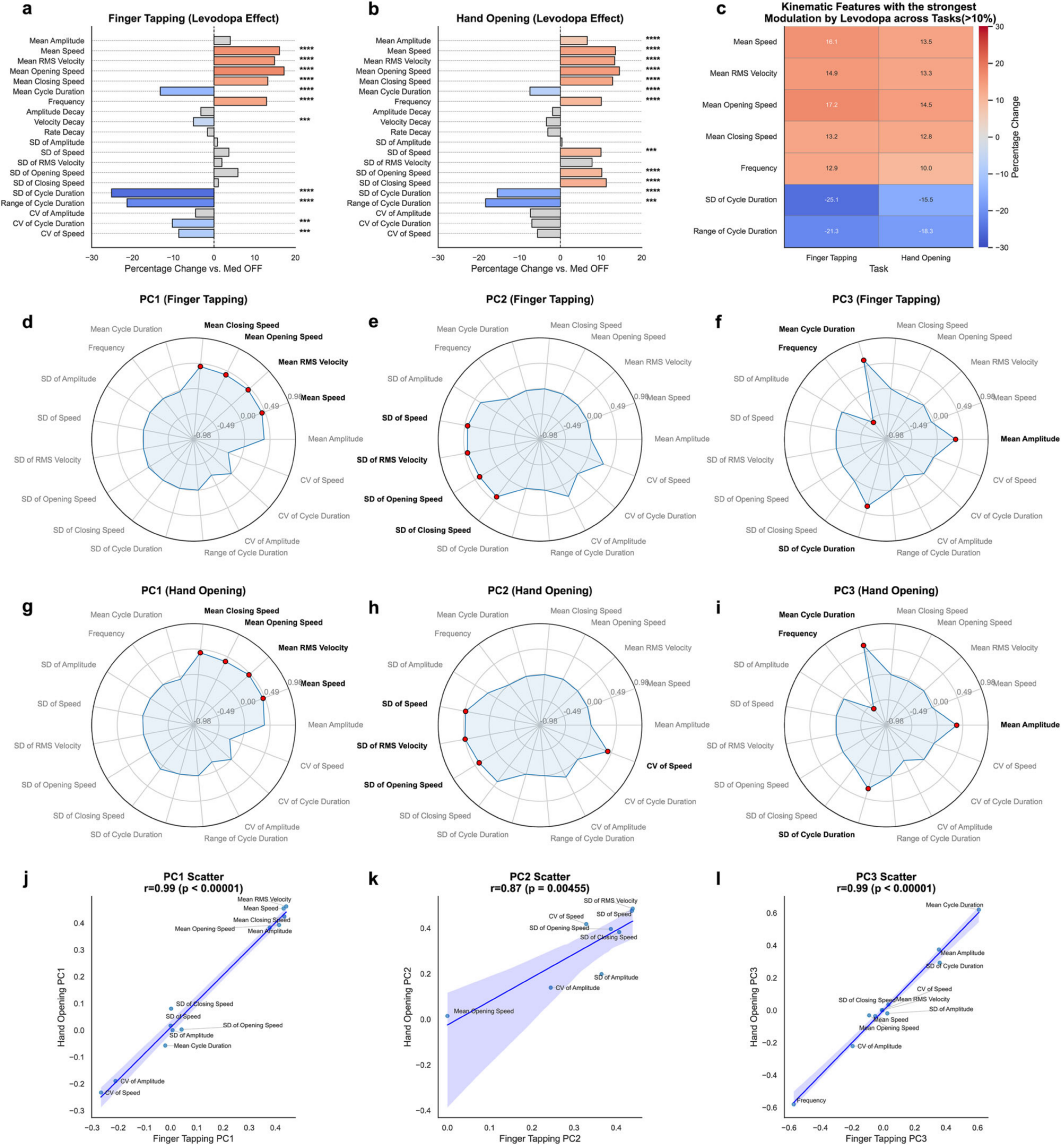
Comprehensive analysis of the medication effects on kinematic variables across tasks. **a**, **b** Horizontal bar charts showing percentage changes in kinematic variables when comparing medication ON (Med ON) to medication OFF (Med OFF) states for finger tapping (**a**) and hand opening (**b**) tasks. Positive changes (red) indicate that levodopa increases movement metrics (e.g., speed, frequency), while negative changes (blue) show reductions (e.g., cycle durations, variability). Solid bars represent significant effects after Bonferroni correction (*p* < 0.0025), demonstrating robust increases in movement speed and decreases in timing variability. Hatched, semi-transparent bars mark non-significant changes, indicating parameters less sensitive to dopaminergic treatment. Asterisks to the right of each bar indicate significance levels: *p < 0.05, **p < 0.01, ***p < 0.001, ****p < 0.0001. These analyses are derived from linear mixed-effects models (LMMs). **c** A heatmap illustrating variables that change by more than 10% across both tasks. This subset underscores levodopa’s consistent enhancement of movement speed-related parameters and its stabilization of movement timing & scale. **d**–**i** Radar plots of the first three sparse principal components (PCs) derived separately for Finger Tapping (**d**–**f**) and Hand Opening (**g**–**i**). Each radar plot highlights the top four highest-loading variables in bold red dots. For Finger Tapping, PC1 (Movement Speed) is exemplified by mean RMS velocity, mean closing speed, mean speed, and mean opening speed, PC2 (Performance Consistency) by standard deviations of RMS velocity, speed, closing speed, and opening speed, and PC3 (Movement Timing & Scale) by mean cycle duration, frequency, standard deviation of cycle duration, and mean amplitude. Similar top-loadings define these domains in Hand Opening, confirming their stability. **j–l** Scatter plots comparing variable loadings for corresponding PCs between the two tasks. Each scatter plot plots the loadings from Finger Tapping on the *x* axis against those from Hand Opening on the *y* axis. A regression line with a 95% confidence interval is fitted to assess the correlation between loadings across tasks. Pearson correlation coefficients (*r*) and their corresponding *p* values are reported in the plot titles. Variables where both loadings are zero are excluded to focus on those with meaningful contributions. High and significant correlations confirm that these three domains are stable and generalize across different motor tasks.

Using linear mixed-effects models (LMMs) that controlled for age and accounted for repeated measures within individuals, we found that levodopa’s effects on motor performance are strikingly selective. Specifically, robust improvements emerged in speed-related parameters (Fig. 1a–c, Table 1) during both finger tapping and hand opening tasks, including significantly increased velocity and faster opening and closing speeds. In contrast, movement amplitude changes were comparatively modest, suggesting that dopaminergic therapy primarily boosts the rapidity and fluidity of movements rather than their magnitude. Additionally, cycle durations shortened, tapping frequencies rose, and variability measures—such as cycle-to-cycle standard deviations—decreased, indicating that levodopa leads to more uniform and stable movement execution.

**Table 1:** Results from linear mixed-effects models: effects of levodopa on kinematic variables during hand opening and finger tapping tasks

Kinematic variable	Percentage change (%) [95% CI] (hand opening)	*P* value (hand opening)	Significant after Bonferroni correction (hand opening)	Percentage change (%) [95% CI] (finger tapping)	*P* value (finger tapping)	Significant after Bonferroni correction (finger tapping)
Mean amplitude	6.59% [3.33% to 9.85%]	7.44e-05	Yes	4.01% [0.42% to 7.59%]	0.0284	No
Mean speed	13.52% [9.89% to 17.14%]	2.85e-13	Yes	16.06% [12.25% to 19.87%]	1.49e-16	Yes
Mean RMS velocity	13.34% [10.01% to 16.68%]	4.52e-15	Yes	14.87% [11.18% to 18.56%]	2.74e-15	Yes
Mean opening speed	14.48% [11.0% to 17.95%]	3.14e-16	Yes	17.21% [13.29% to 21.13%]	7.24e-18	Yes
Mean closing speed	12.80% [8.52% to 17.08%]	4.53e-09	Yes	13.20% [9.46% to 16.95%]	4.66e-12	Yes
Mean cycle duration	−52.23% [−156.58% to 52.12%]	0.327	No	1.37% [−102.09% to 104.84%]	0.979	No
Frequency	10.03% [6.74% to 13.33%]	2.42e-09	Yes	12.90% [9.84% to 15.96%]	1.42e-16	Yes
Amplitude decay	−1.91% [−5.13% to 1.30%]	0.244	No	−3.21% [−6.05% to −0.37%]	0.027	No
Velocity decay	−3.43% [−6.18% to −0.67%]	0.0149	No	−5.04% [−7.58% to −2.50%]	9.88e−05	Yes
Frequency decay	−2.78% [−5.37% to −0.20%]	0.0346	No	−2.43% [−4.89% to 0.02%]	0.052	No
SD of amplitude	0.39% [−4.41% to 5.19%]	0.873	No	0.86% [−3.91% to 5.62%]	0.724	No
SD of speed	9.96% [4.88% to 15.04%]	0.00012	Yes	3.67% [−0.99% to 8.33%]	0.123	No
SD of RMS velocity	7.78% [2.73% to 12.84%]	0.0026	No	1.95% [−2.79% to 6.68%]	0.421	No
SD of opening speed	10.16% [5.41% to 14.92%]	2.80e−05	Yes	5.87% [1.45% to 10.3%]	0.009	No
SD of closing speed	11.26% [6.53% to 16.0%]	3.16e−06	Yes	1.08% [−3.16% to 5.31%]	0.617	No
SD of cycle duration	−15.78% [−21.49% to −10.07%]	6.13e−08	Yes	−25.58% [−32.32% to −18.83%]	1.08e−13	Yes
Range of cycle duration	−18.34% [−28.18% to −8.51%]	2.57e−04	Yes	−21.34% [−30.13% to −12.54%]	2.00e−06	Yes
CV of amplitude	−7.36% [−13.24% to −1.48%]	0.0142	No	−4.53% [−9.72% to 0.67%]	0.088	No
CV of cycle duration	−6.99% [−11.98% to −2.0%]	0.0060	No	−10.24% [−15.68% to −4.81%]	0.00022	Yes
CV of Speed	−5.62% [−10.46% to −0.78%]	0.0227	No	−8.63% [−13.01% to −4.25%]	0.00011	Yes

This table summarizes the effects of levodopa (Med ON) compared to the OFF medication state (Med OFF) on a range of kinematic variables obtained during the Hand Opening and Finger Tapping tasks. For each variable, it displays the percentage change following levodopa administration, the corresponding *p* value, and indicates whether this effect remains statistically significant after Bonferroni correction (*p* < 0.0025). Effect sizes (percentage changes), *p* values, and significance levels were derived from linear mixed-effects models with age as a covariate and a random intercept for each patient.

To better characterize these levodopa-responsive changes, we applied sparse PCA, to identify interpretable dimensions of kinematic variability across both Finger Tapping and Hand Opening tasks. This approach distilled the data into three stable domains—Movement Speed, Consistency, and Movement Timing & Scale—that collectively capture the majority of variance in the data and also how dopaminergic therapy shapes motor behavior (Finger Tapping, Fig. 1d–f; Hand Opening, Fig. 1g–i). The first principal component (PC1) consistently explained the largest portion of the variance and was primarily characterized by high positive loadings for speed-related variables, including mean RMS velocity, mean closing speed, mean speed, and mean opening speed (Fig. 1d, g). We therefore interpret PC1 as representing Movement Speed, reflecting the overall pace and briskness of movement execution, which robustly increased with levodopa.

The second principal component (PC2) was defined by strong positive loadings on variability metrics, particularly the standard deviations (SD) of RMS velocity, speed, closing speed, and opening speed (Fig. 1e, h). This component captures the consistency and uniformity of movements across repeated cycles. We term PC2 Performance Consistency, with levodopa generally leading to changes in these variability metrics.

The third principal component (PC3) showed high loadings primarily for mean cycle duration, frequency (negative loading, consistent with duration), standard deviation of cycle duration, and mean amplitude (Fig. 1f, i). This component captures linked changes in the fundamental timing characteristics and overall scale of the movements. Given these defining features, we interpret PC3 as representing Movement Timing & Scale, aspects also significantly modulated by levodopa.

Notably, Pearson correlation analyses confirmed that the corresponding PCs were highly comparable between Finger Tapping and Hand Opening tasks (Fig. 1j–l, *p* < 0.01 for PC2, *p* < 0.00001 for PC1 and PC3). This strong alignment underscores the stability and generalizability of the three identified domains across different motor tasks. In turn, these results suggest that the fundamental kinematic features enhanced by levodopa are both consistent and robust, capturing core aspects of dopaminergically mediated motor improvements in PD.

Our findings demonstrate the application of a minimally intrusive, video-based, AI-driven approach to quantify motor function in PD. These results show that levodopa significantly increases movement velocity and smoothness, while exerting a comparatively modest influence on movement amplitude. To our knowledge, no previous systematic, controlled studies have thoroughly characterized the kinematic effects of levodopa on PD-related movements at scale. Earlier investigations often relied on specialized, cumbersome measurement techniques—such as magnetic coils or ultrasound markers—making direct comparisons challenging^10,11^. Where such comparisons are possible, our results align with previous observations that levodopa consistently improves speed-related parameters (e.g., angular velocity, movement frequency) but exerts less robust effects on movement amplitude^10,11^. Similarly, our observed gains in movement smoothness parallel earlier reports linking improved fluidity to reduced akinesia^10^. Collectively, this evidence suggests that dopaminergic therapy preferentially enhances certain kinematic features of motor function in PD.

From a clinical perspective, quantifying improvements in speed and consistency using a scalable, video-based approach offers the potential for more precise and objective treatment monitoring. The three identified domains—speed, consistency, and movement timing & scale—offer a potential coherent and structured framework for interpreting dopaminergic treatment effects. Further validation is needed to determine their utility for potentially informing patient-specific therapy adjustments, tracking disease progression, and supporting personalized medicine approaches. By capturing data under real-world conditions and without relying on specialized equipment, our methods aim to mitigate some of the reproducibility challenges encountered in earlier studies^11^. Moreover, current clinical rating scales—often subjective and less granular^12^—commonly fail to detect subtle but meaningful motor improvements, especially in early disease stages^13^. Indeed, recent evidence indicates that even among movement-disorder experts, interrater reliability for Finger Tapping bradykinesia can be only moderate at best, with nearly one in four healthy controls misclassified as having bradykinesia^2^. By contrast, our video-based approach provides objective quantification of amplitude, speed, and variability, potentially reducing these rater-dependent discrepancies while enhancing sensitivity to dopaminergic treatment effects.

Beyond the immediate clinical implications, these kinematic domains offer a scaffold for linking improvements in motor behavior to underlying neural mechanisms. Future research could integrate these kinematic domains with neurophysiological signals (e.g., brain imaging, wearable sensors, neural recordings) to map each domain onto specific neural circuits and oscillatory patterns. Such integrative studies could inform more targeted therapeutic strategies, enabling clinicians to tailor medication regimens or introduce adjunctive therapies aimed at addressing the dominant deficit—be it speed, consistency, or movement timing & scale. Furthermore, these kinematic domains may serve as sensitive endpoints in evaluating novel pharmacological agents or neuromodulation techniques (e.g., adaptive DBS, focused ultrasound), providing more nuanced assessments of therapeutic impact than traditional rating scales.

While this retrospective study and focus on two tasks may limit immediate generalizability, the demonstration of robust, domain-level improvements under real-world conditions provides a template for future investigations. Extending these methods to additional tasks, patient populations, disease severities, and digital intervention paradigms will further clarify their utility. By doing so, we move closer to fully realizing the potential of digital medical interventions: effective, personalized, and grounded in rigorous, real-world evidence.

### Limitations

Several limitations should be considered. First, this study analyzed retrospective data collected over 13 years, introducing potential heterogeneity in recording conditions and protocols. Second, our findings are based on two specific hand tasks (Finger Tapping, Hand Opening), and generalizing the identified kinematic domains (Speed, Consistency, Movement Timing & Scale) to other PD motor symptoms requires further study. Third, markerless pose estimation, while scalable, may have lower precision than marker-based systems, impacting kinematic feature accuracy. Fourth, the three Sparse PCA components explained 60-70% of the ON-OFF difference variance, meaning other levodopa-responsive features might be less represented in our domain analysis. Finally, while demonstrating robust group-level effects, the direct translation to routine clinical practice necessitates further prospective validation to confirm feasibility, reliability, and clinical utility for individual patient management. Despite these points, our work provides a foundation for more objective, large-scale analysis of motor function in PD.

In summary, our video-based analysis reveals that levodopa primarily enhances movement speed and consistency in PD, with comparatively smaller effects on amplitude. By extracting stable, task-agnostic domains of motor improvement, we establish a conceptual framework that may help guide more nuanced clinical decision-making, could potentially support personalized treatment strategies, and may inform future research aimed at linking motor behavior changes to underlying neural mechanisms.

## Methods

### Patient cohort

Inclusion criteria required a diagnosis of isolated PD, sufficient video quality (≥30 fps). The study was conducted in accordance with the Declaration of Helsinki and was approved by the Medical Ethics Committee at the Julius Maximilian University of Würzburg (Medizinische Ethikkommission an der Julius-Maximilians-Universität Würzburg) (Reference number: 100/24). All participants provided written research consent. This study included 154 patients (123 males and 31 females), who collectively contributed 1242 video recordings. Each patient participated in an average of 4.42 visits (SD = 2.39), with a mean follow-up duration of 526.86 days (SD = 457) since the first visit. The cohort predominantly consisted of middle-aged to older adults, exhibiting a mean age of 61.01 years at their first visit (SD = 7.8 years, ranging from 43 to 78 years). Notably, there were no patients under 40 years or over 80 years at the initial visit. Medication conditions were evenly distributed across the two primary tasks analyzed. In the Finger Tapping task, there were 344 recordings in the OFF medication state and 336 recordings in the ON medication state. Similarly, the Hand Opening task comprised 287 OFF and 275 ON recordings, demonstrating a balanced representation of medication conditions across both tasks. All participants underwent assessments in defined medication ‘OFF’ and ‘ON’ states as part of their clinical evaluation protocol. Detailed baseline demographics, disease characteristics including H&Y scores and Medication response for the cohort are provided in Supplementary Table 2.

### Video recordings, kinematic analysis and outcome measurements

Assessments were performed under standardized medication conditions to evaluate levodopa response. To achieve a defined medication ‘OFF’ state, a strict withdrawal protocol was implemented based on the patient’s usual medication regimen. This involved an overnight washout of at least 12 hours for standard dopaminergic medications. For patients prescribed long-acting formulations (e.g., extended-release dopamine agonists like pramipexole), medication was withdrawn for a minimum of 72 hours prior to the assessment to ensure the best possible true medication-free state. The OFF state video recording was conducted in the morning, typically around 8:00 AM, before the administration of any dopaminergic medication. Following the OFF state recording, patients received a standardized oral levodopa challenge to assess their ‘ON’ state. This consisted of 1.5 times their usual individual morning levodopa equivalent dose, administered using a soluble levodopa/benserazide preparation (Madopar® LT) to facilitate rapid and predictable absorption. The ON state video recording was performed precisely 60 minutes after the intake of the levodopa challenge dose, targeting the expected time of peak clinical effect.

Videos, typically around 30 minutes in length, followed the standardized MDS-UPDRS III protocol. Recordings were made using various camera setups over the years, all producing videos at a minimum of 30 frames per second (fps). For this study, only Finger Tapping and Hand Opening tasks were analyzed, as those have been validated sufficiently^9^.

We developed and utilized VisionMD, an open-source software intended specifically for the video-based kinematic analysis of motor functions in patients with movement disorders. We have previously detailed processes, algorithms, and performance characteristics^9,14^. Briefly, the tool provides a semi-automated pipeline for the analysis of movement tasks. VisionMD operates by first detecting and localizing the subject in the video using an object localization algorithm based on the YOLO architecture^15^, which identifies the position of the subject within each frame. Once the subject is localized, users can define specific tasks to be analyzed by selecting regions of interest in a waveform display. The system allows for frame-level precision in task selection, and predefined motor tasks can be selected in the video.

Once the tasks are defined, VisionMD uses Google’s MediaPipe^16^ markerless pose estimation framework to track the relevant body landmarks, such as finger or hand movements. The software extracts time-series data corresponding to the movement, such as the Euclidean distances between body landmarks, and generates a range of kinematic features, including amplitude, speed, decay parameters, and rhythm metrics. All analyses were conducted locally, ensuring data privacy and security. The kinematic features analyzed are explained in detail in Table 2. We relied on the same set of kinematic features that have been successfully employed and validated in earlier studies using this framework^6,9^.

**Table 2:** Explanation and clinical correlates of kinematic variables in Parkinson’s disease

Feature	Clinical Interpretation
Mean amplitude	Average extent of movement, a decrease suggests hypokinesia
Mean speed	Overall movement quickness; low values may suggest bradykinesia.
Mean RMS velocity	Average velocity of movement; low values may suggest bradykinesia.
Mean opening speed	Speed of fingers or hand opening; decreased in bradykinesia.
Mean closing speed	Speed of fingers or hand closing; decreased in bradykinesia.
Mean cycle duration	Duration of a complete movement cycle; longer durations suggest bradykinesia.
Frequency	Frequency of repetitive movements, with lower rates potentially indicating bradykinesia.
SD amplitude	Variability in movement amplitude, indicating inconsistency in motor control.
SD speed	Variability in speed, reflecting inconsistency in movement quickness.
SD RMS velocity	Variability in RMS velocity, showing fluctuation in movement velocity.
SD opening speed	Variability in opening speed, indicating inconsistent speed during extension movements.
SD Closing speed	Variability in closing speed, showing inconsistent speed during movements.
SD cycle duration	Variability in cycle duration, indicating inconsistent timing in task performance.
Range cycle duration	The difference between the longest and shortest cycles, reflecting inconsistency in movement execution.
Amplitude decay	Reduction in movement amplitude over time, indicative of a sequence effect
Velocity decay	Decrease in movement speed over time, indicative of a sequence effect
Frequency decay	Reduction in movement rate over time, indicative of a sequence effect
CV amplitude	Coefficient of variation for amplitude, indicating relative variability of amplitude.
CV cycle duration	Coefficient of variation for cycle duration, showing relative variability in movement timing.
CV speed	Coefficient of variation for speed, reflecting relative consistency of movement speed.
CV RMS velocity	Coefficient of variation for RMS velocity, indicating variability in overall movement velocity.
CV opening speed	Coefficient of variation for opening speed, showing consistency in speed.
CV closing speed	Coefficient of variation for closing speed, reflecting consistency in speed.

This table shows the kinematic variables measured in PD patients during motor task assessments and outlines their clinical interpretations. It serves as a guide to understanding these variables from a clinical perspective, linking features such as Mean Speed and Mean Amplitude to symptoms like bradykinesia and hypokinesia, respectively. The interpretations provided in the ‘Clinical Interpretation’ column are intended to aid the reader by connecting the measured kinematic variables to established clinical concepts and phenomena relevant to Parkinson’s disease motor function (such as bradykinesia, hypokinesia, variability, and sequence effects, as reviewed in ref. ^1^). These serve as a general guide to the potential meaning of each feature within a clinical context.

### Statistical analyses

We conducted statistical analyses using LMMs to evaluate the effects of levodopa on kinematic variables. Age at Visit was included as a covariate to adjust for age-related effects. A random intercept for each patient was included to account for repeated measurements across multiple visits. Outliers were excluded by removing data points exceeding the 99th percentile for each kinematic variable.

To identify effect sizes for the change between the two medication conditions, we extracted the fixed-effect coefficients corresponding to the condition levels (e.g., Med ON vs. OFF) for each kinematic variable, and their associated p-values. To interpret the practical significance of the condition effects, we calculated the percentage change in each kinematic variable due to the condition using the formula:

1 $${Percentage\; Change}\left( \% \right)=\left(\frac{{Coefficient}}{{Baseline\; Mean}}\right)\,x\,100$$

To identify underlying dimensions of movement variability responsive to levodopa, we applied Sparse PCA to the standardized differences between ON-OFF difference data derived from the full set of 20 kinematic variables described in Table 2, including amplitude, speed, variability, timing, and decay parameters. We selected Sparse PCA over standard PCA for several reasons. Standard PCA typically produces components that are linear combinations of all original features, which can make interpreting the biological or clinical meaning of the components difficult, particularly with the moderate number of kinematic variables (*n* = 20) used here. Sparse PCA, through L1 regularization, forces many of the component loadings to be exactly zero^17,18^. This yields components defined by smaller, distinct subsets of variables, significantly enhancing interpretability and facilitating the identification of potentially distinct kinematic dimensions impacted by levodopa^17^. The analysis was performed on the calculated difference scores (ON state value–OFF state value) for each kinematic variable, which were then standardized (zero mean, unit variance) prior to applying Sparse PCA using scikit-learn’s Sparse PCA function^19^ (alpha parameter set to 1).

To determine the optimal number of principal components (PCs) for analysis, we examined the cumulative explained variance of Sparse PCA components. The ‘elbow point’ of the cumulative variance plot indicated that the first three components captured the majority of variance in the data, with minimal contribution from additional components (See Supplementary Fig. 1). Based on this, we retained three PCs for further analysis and visualization. To assess whether the identified kinematic domains (represented by the PCs) were consistent across different motor tasks, we evaluated the stability of the component loadings between the Finger Tapping and Hand Opening tasks. Sparse PCA was run independently on the standardized ON-OFF difference data from each task. We then compared the loading vectors of the first three corresponding components (PC1, PC2, PC3) across the two tasks. For each pair of corresponding components (e.g., PC1-Finger Tapping vs. PC1-Hand Opening), we computed the Pearson correlation coefficient between their loading vectors. This correlation was calculated across all kinematic variables, excluding only those variables that had a zero loading for that specific component in *both* tasks. High, significant positive correlations (as shown in Fig. 1j–l) indicate that the same kinematic features contribute similarly (in direction and relative magnitude) to the corresponding components in both Finger Tapping and Hand Opening. This suggests that the underlying dimensions of levodopa-responsive change captured by these components are stable and generalize across these two upper-limb motor tasks.

To further investigate the components beyond the first three identified domains, we examined the loadings and cross-task stability of the fourth and fifth PCs derived from the Sparse PCA on standardized medication difference scores (Supplementary Fig. 2). Panels S2a (Finger Tapping PC4), S2b (Finger Tapping PC5), S2d (Hand Opening PC4), and S2e (Hand Opening PC5) show that PC4 and PC5 capture mixed combinations of kinematic features, often related to variability (e.g., cycle duration SD/CV, speed CV, amplitude CV) and scale (mean speed/amplitude), without representing single, clearly interpretable domains like the initial three components. Crucially, the correlation of loadings between Finger Tapping and Hand Opening was weak and non-significant for both PC4 (*r* = −0.48, *p* = 0.194; Panel S2c) and PC5 (*r* = −0.48, *p* = 0.281; Panel S2f). This contrasts sharply with the high stability observed for PC1, PC2, and PC3 (*r* > 0.85, *p* < 0.01; Fig. 1j–l). The lack of generalizability for PC4 and PC5 across tasks supports our focus on the first three components as representing the most robust and stable dimensions of levodopa-responsive change in this dataset, consistent with the elbow observed in the explained variance analysis (Supplementary Fig. 1)”.

All analyses were conducted using the statsmodels Python package (version 0.14.2). The LMMs were implemented using the MixedLM function. Data manipulation and preprocessing were performed using pandas (version 2.2.2) and numpy (version 1.26.4). A Bonferroni correction was applied, adjusting the significance threshold to *p* < 0.0025 (i.e., 0.05/20) to account for multiple comparisons.

## Supplementary information

Supplementary materials

## Data Availability

The raw video data analyzed in this study contains sensitive patient information and cannot be made publicly available due to privacy regulations and institutional policies. However, the derived kinematic features data matrix used for all statistical analyses and figure generation in this manuscript is publicly available in the same GitHub repository as the analysis code: [https://github.com/Flolan2/VisionSC_med].

## Code availability

The core VisionMD computer vision pipeline used for feature extraction is open-source and available for download at https://www.visionmd.ai/. The specific Python code used to perform the statistical analyses (including LMMs, Sparse PCA) and generate the figures presented in this manuscript is publicly available in a GitHub repository at: [https://github.com/Flolan2/VisionSC_med].
