# Supplementary material for: Computer vision uncovers three fundamental dimensions of levodopa-responsive motor improvement in Parkinson’s disease
Source: NPJ Parkinsons Dis. 2025 May 28;11:140. doi: 10.1038/s41531-025-00999-w (PMC12119790; doi:10.1038/s41531-025-00999-w)
Supplement: Supplementary file 1 — Supplementary materials [file 41531_2025_999_MOESM1_ESM.pdf]

Computer vision uncovers three fundamental dimensions of levodopa-responsive motor improvement in Parkinson's disease - Supplementary Information

Supplementary Figure: PCA Explained Variance and Scree Plots

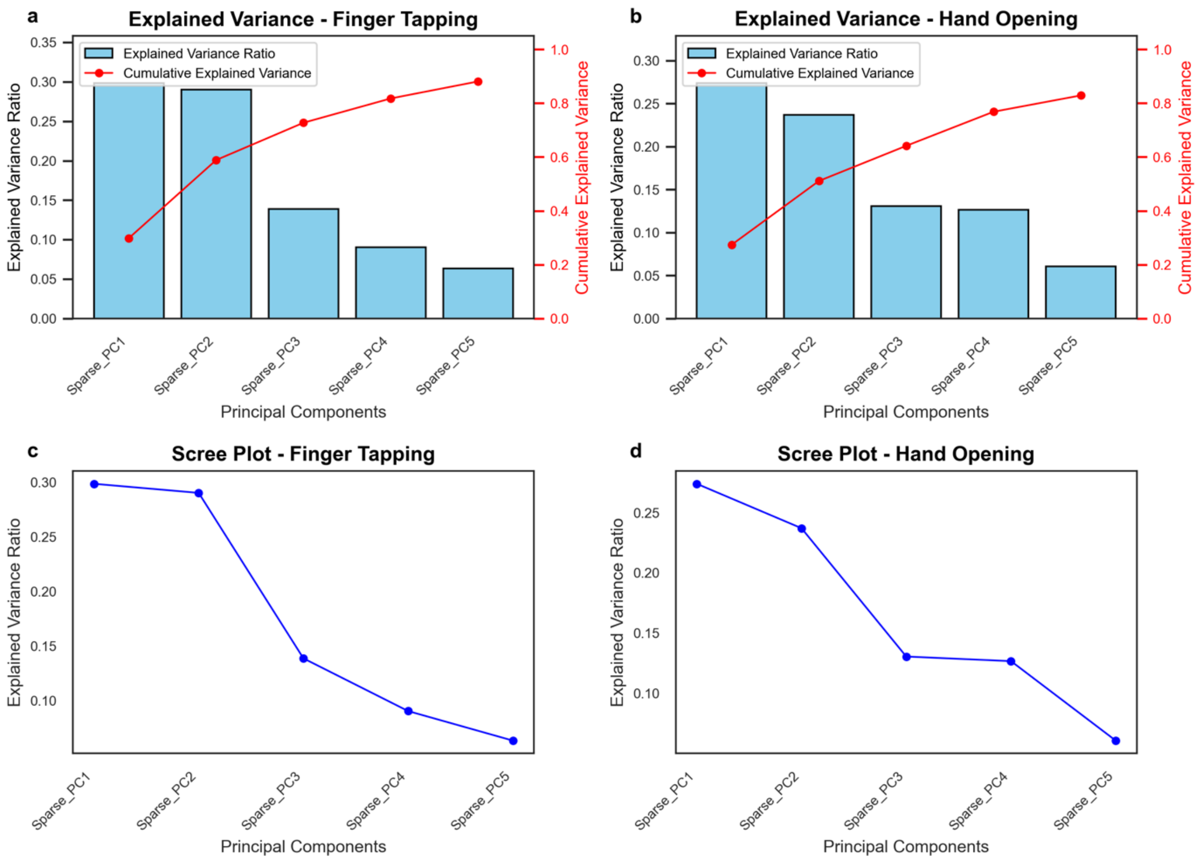

Supplementary Figure 1: PCA Explained Variance and Scree Plots for Finger Tapping and Hand Opening Tasks

(a, b) Bar plots illustrate the proportion of variance explained by individual principal components (PCs) for the Finger Tapping (a) and Hand Opening (b) tasks. Blue bars represent the explained variance ratio of each PC, while the red circles and line represent the cumulative explained variance. **(c, d)** Scree plots for the Finger Tapping (c) and Hand Opening (d) tasks. Each point corresponds to a PC, plotted in order of descending explained variance ratio. The "elbow" in the plot indicates a natural cutoff point beyond which additional PCs contribute relatively little to the total explained variance. By visualizing this pattern, the scree plots provide a complementary, intuitive approach to identifying the number of meaningful PCs to consider.

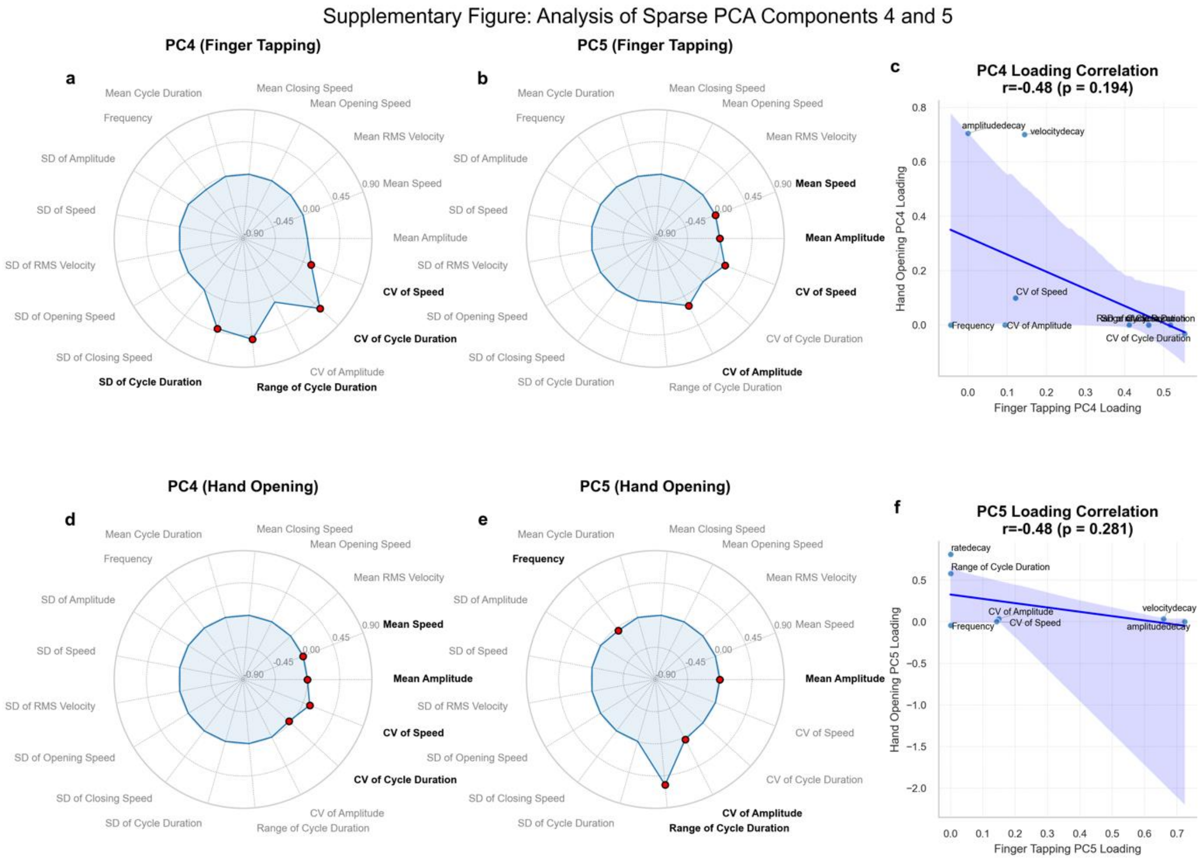

Supplementary Figure 2: Analysis of Sparse PCA Components 4 and 5: Loadings and Cross-Task Stability

This figure details the analysis of the fourth (PC4) and fifth (PC5) principal components derived from Sparse Principal Component Analysis (Sparse PCA) performed on standardized ON-OFF medication difference scores for the Finger Tapping and Hand Opening tasks. **(a, b, d, e)** Spider plots illustrating the loadings of kinematic variables onto PC4 (a, d) and PC5 (b, e) for Finger Tapping (a, b) and Hand Opening (d, e). Each plot shows the contribution of different features to the component. Red dots highlight the top four variables with the highest absolute loading values for that specific component and task. The radial axis represents the loading value, scaled consistently across PC4 and PC5 plots based on the maximum absolute loading observed for these components. **(c, f)** Scatter plots comparing the variable loadings for PC4 (c) and PC5 (f) between the Finger Tapping (x-axis) and Hand Opening (y-axis) tasks. Each point represents a kinematic variable. The regression line (blue) with its 95% confidence interval (shaded area) is shown. Pearson correlation coefficients (r) and p-values indicate a lack of significant correlation between the loading patterns across tasks for both PC4 ($r = -0.48$, $p = 0.194$) and PC5 ($r = -0.48$, $p = 0.281$).

Kinematic Variable	Age Coefficient [95% CI] (Hand Opening)	Age P-Value (Hand Opening)	Age Coefficient [95% CI] (Finger Tapping)	Age P-Value (Finger Tapping)
**Mean Amplitude**	-0.003 [-0.005, -0.001]	0.005	0.001 [-0.005, 0.006]	0.763
**Mean Speed**	0.001 [0.000, 0.001]	0.003	0.001 [-0.000, 0.002]	0.219
**Mean RMS Velocity**	-0.011 [-0.024, 0.001]	0.077	0.008 [-0.027, 0.044]	0.641
**Mean Opening Speed**	0.003 [-0.000, 0.006]	0.065	0.003 [-0.004, 0.010]	0.340
**Mean Closing Speed**	-0.007 [-0.018, 0.003]	0.178	-0.000 [-0.030, 0.029]	0.990
**Mean Cycle Duration**	0.006 [0.002, 0.011]	0.003	0.001 [-0.004, 0.007]	0.638
**Frequency**	-0.011 [-0.023, 0.001]	0.067	0.013 [-0.018, 0.044]	0.417
**Amplitude Decay**	0.002 [-0.002, 0.006]	0.291	0.004 [-0.002, 0.010]	0.196
**Velocity Decay**	0.094 [-0.065, 0.254]	0.247	-0.043 [-0.214, 0.128]	0.623
**Frequency Decay**	0.001 [-0.000, 0.002]	0.082	0.000 [-0.000, 0.001]	0.491
**SD of Amplitude**	0.008 [0.001, 0.014]	0.017	0.002 [-0.001, 0.006]	0.147
**SD of Speed**	0.001 [-0.002, 0.003]	0.710	-0.001 [-0.005, 0.002]	0.522
**SD of RMS Velocity**	-0.000 [-0.003, 0.003]	0.912	-0.002 [-0.005, 0.001]	0.176
**SD of Opening Speed**	-0.000 [-0.002, 0.002]	0.940	-0.000 [-0.003, 0.002]	0.679
**SD of Closing Speed**	0.003 [0.001, 0.006]	0.002	0.000 [-0.001, 0.002]	0.727
**SD of Cycle Duration**	0.002 [0.001, 0.004]	0.007	0.001 [-0.000, 0.002]	0.082
**Range of Cycle Duration**	0.003 [-0.007, 0.013]	0.593	-0.001 [-0.014, 0.012]	0.884
**CV of Amplitude**	-0.005 [-0.010, 0.000]	0.059	0.002 [-0.013, 0.017]	0.759
**CV of Cycle Duration**	0.001 [-0.000, 0.003]	0.108	0.001 [-0.002, 0.004]	0.342
**CV of Speed**	0.003 [0.001, 0.005]	0.001	-0.000 [-0.001, 0.001]	0.856

Supplementary Table 1: Linear Mixed-Effects Model Results for Age Effects on Kinematic Variables

This table presents the results from Linear Mixed-Effects Models (LMMs) detailing the effect of Age at Visit on kinematic variables, analyzed separately for Hand Opening and Finger Tapping tasks. The LMM formula controlled for medication state (Med ON vs. Med OFF) and included a random intercept for Patient_ID: Outcome ~ C(Condition_MED, Treatment(reference='OFF')) + Age_at_Visit. The 'Age Coefficient [95% CI]' shows the estimated change in the outcome variable (and its 95% Confidence Interval) per one-year increase in age at visit. P-values are shown in scientific notation and are rounded to three decimal places. CI = Confidence Interval.

Characteristic	***Value***
Total Patients	154
Sex	
Male	123 (79.9%)
Female	31 (20.1%)
Age at Assessment (years; n=154)	
Mean ± SD	61.7 ± 9.2
Range [min – max]	41.7 – 82.8
Hoehn & Yahr Stage (n=151)	
Stage 2	24 (15.9%)
Stage 3	110 (72.8%)
Stage 4	16 (10.6%)
Stage 5	1 (0.7%)
MDS-UPDRS Part III Motor Score	
OFF Medication (n=151)	

Mean ± SD	39.4 ± 14.4
Range [min – max]	4 – 87
ON Medication (n=154)	
Mean ± SD	17.8 ± 11.1
Range [min – max]	0 – 53
Absolute Improvement (OFF-ON; n=151)_¹	
Mean ± SD	21.6 ± 12.4
Range [min – max]	-8 – 69
Percentage Improvement (%; n=151)_¹	
Mean ± SD	54.7% ± 24.6%
Range [min – max]	-15.4% – 100.0%

Supplementary Table 2: Baseline Demographic and Clinical Characteristics of the Patient Cohort

This table summarizes the baseline characteristics of the 154 Parkinson's disease patients included in the study, based on data available at a primary assessment visit. Clinical scores (MDS-UPDRS Part III Motor Score, Hoehn & Yahr stage) were recorded at this visit. Descriptive statistics are presented as mean ± standard deviation (SD) and range [min – max] or as counts (N) and percentages (%). Calculations for specific metrics exclude patients with missing data for that metric.
